# Emergence of *cfr*-Mediated Linezolid Resistance in *Staphylococcus* *aureus* Isolated from Pig Carcasses

**DOI:** 10.3390/antibiotics9110769

**Published:** 2020-11-02

**Authors:** Hee Young Kang, Dong Chan Moon, Abraham Fikru Mechesso, Ji-Hyun Choi, Su-Jeong Kim, Hyun-Ju Song, Mi Hyun Kim, Soon-Seek Yoon, Suk-Kyung Lim

**Affiliations:** Bacterial Disease Division, Animal and Plant Quarantine Agency, 177 Hyeksin 8-ro, Gimcheon-si, Gyeongsangbuk-do 39660, Korea; kanghy7734@korea.kr (H.Y.K.); ansehdcks@korea.kr (D.C.M.); abrahamf@korea.kr (A.F.M.); wlgus01@korea.kr (J.-H.C.); kimsujeong27@gmail.com (S.-J.K.); shj0211@korea.kr (H.-J.S.); kimmh940301@naver.com (M.H.K.); yoonss24@korea.kr (S.-S.Y.)

**Keywords:** carcass, *cfr* gene, *fexA* gene, linezolid, mutation, pig, public health, *S. aureus*

## Abstract

Altogether, 2547 *Staphylococcus aureus* isolated from cattle (*n* = 382), pig (*n* = 1077), and chicken carcasses (*n* = 1088) during 2010–2017 were investigated for linezolid resistance and were further characterized using molecular methods. We identified linezolid resistance in only 2.3% of pig carcass isolates. The linezolid-resistant (LR) isolates presented resistance to multiple antimicrobials, including chloramphenicol, clindamycin, and tiamulin. Molecular investigation exhibited no mutations in the 23S ribosomal RNA. Nevertheless, we found mutations in ribosomal proteins rplC (G121A) and rplD (C353T) in one and seven LR strains, respectively. All the LR isolates carried the multi-resistance gene *cfr*, and six of them co-carried the *mecA* gene. Additionally, all the LR isolates co-carried the phenicol exporter gene, *fexA*, and presented a high level of chloramphenicol resistance. LR *S. aureus* isolates represented 10 genotypes, including major genotypes ST433-t318, ST541-t034, ST5-t002, and ST9-t337. Staphylococcal enterotoxin and leukotoxin-encoding genes, alone or in combination, were detected in 68% of LR isolates. Isolates from different farms presented identical or different pulsed-field gel electrophoresis patterns. Collectively, toxigenic and LR *S. aureus* strains pose a crisis for public health. This study is the first to describe the mechanism of linezolid resistance in *S. aureus* isolated from food animal products in Korea.

## 1. Introduction

Linezolid belongs to the oxazolidinone antibiotics and is approved for the treatment of severe infections caused by multidrug-resistant Gram-positive pathogens including methicillin-resistant *Staphylococcus aureus* (MRSA) and vancomycin-resistant enterococci [1]. Linezolid interferes with the peptidyltransferase site of the bacterial ribosome. This leads to disruption of protein synthesis and inhibition of bacterial growth [2]. However, the emergence of linezolid-resistant (LR) staphylococci and enterococci poses a significant and interdisciplinary public health challenge [1].

Mutation in the central loop of domain V of 23S ribosomal ribonucleic acid (rRNA) (C2161T) is the primary mechanism of linezolid resistance. However, redundancy of rRNA genes makes it difficult to reach sufficient levels of resistance by a mutation in a single allele [3]. In addition, rRNA mutations often negatively affect ribosome functions and are rapidly reversed in the absence of selection [4]. Therefore, the resistance mechanism based on chemical modification of rRNA such as the acquisition of the multi-resistance gene *cfr* is more common [5]. Linezolid resistance is also associated with mutations in the genes coding for ribosomal proteins (L3 and L4). Moreover, the novel *optrA* and *poxtA* genes have been implicated in transferrable linezolid resistance [1,6].

The *cfr* gene was initially described in a bovine *Staphylococcus sciuri* isolate [7]. It catalyzes the methylation of A2503 in the 23S rRNA of the large ribosomal subunit [8]. The methylation leads to cross-resistance against several antimicrobial classes of drugs (phenicols, lincosamides, pleuromutilins, macrolides, oxazolidinones, and streptogramin A), conferring multidrug resistance [9,10]. Therefore, these antimicrobials can mediate selective pressure in favor of the *cfr* gene. The *cfr* gene was mostly identified on plasmids, often in close proximity to insertion sequences, which play a vital role in the mobility of the *cfr* gene [11]. These mobile structures have been detected among several Gram-positive and Gram-negative bacteria, including bacteria other than staphylococci, *Enterococcus faecalis*, and *Escherichia coli* [9].

The occurrence of LR *S. aureus* in humans and food animals has been increasingly reported in many countries [6,12,13,14,15]. Previous studies in South Korea (Korea) demonstrated the occurrence of linezolid resistance in *S. aureus* strains recovered from hospital patients [16,17], pigs, and chicken carcasses [18]. Despite a single report on the occurrence of the *cfr* gene in MRSA recovered from pig carcasses [19], to our knowledge, no attempt has been made on the detailed mechanism of linezolid resistance among *S. aureus* isolates recovered from animal sources in Korea to date. Korea’s meat consumption has increased in the past few years, with pork remaining the most popular source. Thus, continuous surveillance on the emergence of antimicrobial-resistant bacterial strains in animal carcasses is essential to safeguard public health. In this study, we aimed to determine the occurrence of linezolid resistance in *S. aureus* isolated from major food animal carcasses from 2010 to 2017, as well as to study the underlying mechanism(s) of resistance.

## 2. Results and Discussion

### 2.1. Prevalence and Antimicrobial Susceptibility Profiles of LR S. aureus

Linezolid resistance was detected in 2.3% of *S. aureus* isolated from pigs (Table 1). The low linezolid resistance rate among pig isolates in this study was consistent with those reported in Korea in 2011 (2.9%) [18] and 2015 (0.14%) [19], but lower than a recent report in South Africa (14%) [14]. Similarly, *S. aureus* isolated from medical centers in various countries presented very low linezolid resistance rates (≈1%) [13,20,21]. Agreeing with our recent report [19], resistance was not observed among cattle and chicken isolates. In contrast, previous studies in Korea [18] and South Africa [14] reported that 1.2% and 9% of chicken and cattle carcass isolates, respectively, were resistant to linezolid. Linezolid is not approved for animal use in Korea. Thus, the observed difference in resistance could be associated with the frequent use of phenicols, pleuromutilins, and lincosamides in the Korean pig industry, which might co-select resistance to linezolid [22]. The detection of LR *S. aureus* strains in pig carcasses is worrisome because of the potential transmission to humans through the food supply chain.

### 2.2. Mutations and Antimicrobial Resistance Genes

Spontaneous mutation in the multiple copies of 23S rRNA alleles is the primary mechanism of linezolid resistance [23]. None of the identified LR isolates exhibited this type of mutation. Resistance mediated by mutations in the 23S rRNA appears rarely, develops slowly, and is not transferrable between bacterial species [24]. However, all of the identified LR isolates carried the *cfr* gene (Table 2), which is associated with low-level linezolid resistance [6]. Previous studies have also identified *cfr*-harboring *S. aureus* mainly from humans and to a lesser extent from food animals in various countries, including Korea [15,19,25,26]. Notably, all but two of the *cfr*-carrying isolates were from different farms. The extensive dissemination of *cfr*-carrying strains among pig farms could be related to the association of the *cfr* gene to mobile elements [9], which facilitates the mobilization and horizontal transfer [27]. Moreover, the low fitness cost could attribute to the wide dissemination of the *cfr* gene. Previous studies have demonstrated that genes that come at low cost can stably persist in the cells, even when pathogens were not exposed to antibiotics [27,28,29,30].

Linezolid resistance mediated by the *cfr* gene has also been shown to coexist with other resistance mechanisms [17]. We identified mutations in ribosomal proteins rplC (G121A) and rplD (C353T) in one and seven LR strains, respectively (Table 2). These types of mutations have been linked with resistance or decreased susceptibility to linezolid [31]. The difference in linezolid resistance mechanisms between human isolates, mutations in the 23S rRNA gene [17], and pig isolates in this study indicates the presence of unique clones in the pig industry.

All the identified *cfr*-carrying isolates were resistant to multiple antimicrobials including chloramphenicol, clindamycin, and tiamulin, and co-carried phenicol exporter gene *fexA* (Table 2). The *cfr* gene has been reported to confer resistance to antimicrobials that are widely used in veterinary medicine, such as macrolides, tetracyclines, phenicols, and lincosamides [5]. Previous studies have also shown the co-existence of the *cfr* gene and other resistance genes, which facilitates its co-selection and spread [26,32]. Moreover, six of the LR strains co-carried the *mecA* gene. The co-existence of the *mecA* and *cfr* genes is an unwelcome development because linezolid is among the last resort of antimicrobial agents against MRSA in humans.

### 2.3. Molecular Characteristics of LR S. aureus Isolates

The potential risk of transmission of *cfr*-carrying *S. aureus* between pigs and humans is a growing health concern. In this study, the 25 LR-isolates belonged to ST433-t318 (*n* = 6); ST541-t034 (*n* = 6); ST5-t002 (*n* = 4); ST9-t337 (*n* = 3); and each of ST5-t548, ST9-t899, ST398-t034, ST398-t1170, ST433-t021, and ST2007-t8314. Five of these lineage types (ST9, ST398, ST433, ST541, and ST2007) were livestock-associated (LA) strains, while ST5 *S. aureus* was the only human-associated (HA) strain. Except for ST2007, all the LA and HA strains were reported in pigs and farmers in Korea [19,33,34], indicating the possibility of transmission between pigs and humans. Korea is one of the markets with the fastest growing consumption of pork in the world. Hence, the emergence of *cfr*-carrying *S. aureus* with unique molecular characteristics in pig carcasses is concerning.

We identified LR *S. aureus* strains with sequence type (ST2007) and spa types (ST5-t548 and ST433-t318) that had not been reported in Korea, suggesting the emergence of new clones that carried the *cfr* gene and/or have mutations in ribosomal protein rplD. Although the linezolid resistance profiles are unknown, the ST5-t548 [35], ST433-t318 [36], and ST2007-t8314 [37] strains were detected in humans and/or pigs in China, Poland, and the United States, respectively. Moreover, we observed LR-ST398 *S. aureus* carrying a novel spa type (t1170) in farm GG-5, suggesting an evolutionary change in *S. aureus*.

Staphylococcal enterotoxin and leukotoxin-encoding genes, alone or in combination, were detected in 68% of LR isolates: *seg* (28%, 7/25), *seg*-*sei*-*sem*-*sen*-*seo* (24%, 6/25), and *seg*-*sei*-*sem*-*sen*-*seo*-*lukED* (16%, 4/25) (Table 2). Eight (32%) isolates, including the five MRSA strains, were negative for any of the tested virulence factor genes. In agreement with Price et al. [38], the HA-ST5 strains appeared to be more virulent than the LA strains. Additionally, multiple virulence factor genes were detected in one of the LA strains, ST9. *S. aureus* harboring the classical enterotoxins and leucotoxins can spread to humans either through contact or via the food chain and are capable of causing food-related illnesses in humans [39].

Analysis using pulsed-field gel electrophoresis (PFGE) revealed four distinct PFGE types, with identical PFGE types in isolates belonging to the same sequence types (Figure S1). Isolates from different farms in the same or different provinces presented identical or different PFGE patterns. These results might suggest cross-contamination in the slaughterhouse, or clonal dissemination and/or persistence of specific clones among farms, not only within a province but also in different provinces.

## 3. Materials and Methods

### 3.1. Sample Collection and Isolation of S. aureus

A total of 2547 *S. aureus* isolates (382 cattle, 1077 pig, and 1088 chicken carcass isolates) were obtained from 16 laboratories/centers participating in the Korean Veterinary Antimicrobial Resistance Monitoring System. Sample collection and isolation of *S. aureus* were performed as described previously [19]. Briefly, the back and chest of cattle and pig carcasses were swabbed with sterile gauze pads wetted with buffered peptone water (BPW) (Becton Dickinson, Sparks, MD, USA), while the whole carcasses of chickens were rinsed in Phosphate Buffered Water (PBW). Homogenized samples were inoculated into tryptic soy broth (Becton Dickinson) containing 6.5% sodium chloride and incubated at 37 ℃ for 16 h. Following incubation, one or two loops from each enrichment broth were streaked onto mannitol salt agar (Difco, Detroit, MI, USA). Suspected colonies were then identified by matrix-assisted laser desorption/ionization time-of-flight mass spectrometry (Biomerieux, Marcy L’Etoile, France). *S. aureus* and MRSA isolates were further confirmed by a multiplex polymerase chain reaction (PCR) assay specific for the *16S rRNA*, *clfA*, and *mecA* genes [40].

### 3.2. Antimicrobial Susceptibility Testing and Detection of Resistance Genes

Linezolid susceptibility was determined by the broth dilution method [41], using linezolid-containing plates (1–8 µg/mL) (EUST, TREK Diagnostics Systems, Cleveland, OH). The LR isolates were screened for the presence of *cfr*, *fexA*, *optrA*, and *poxtA* genes using PCR [6,42]. The susceptibility profiles of the identified LR isolates were further evaluated for the following 19 antimicrobial agents using antibiotic-containing plates (EUST, TREK Diagnostics Systems, Cleveland, OH): cefoxitin (0.5–16 µg/mL), chloramphenicol (4–64 µg/mL), ciprofloxacin (0.25–8 µg/mL), clindamycin (0.12–4 µg/mL), erythromycin (0.25–8 µg/mL), fusidic acid (0.5–4 µg/mL), gentamicin (1–32 µg/mL), kanamycin (4–64 µg/mL), mupirocin (0.5–256 µg/mL), penicillin (0.12–2 µg/mL), quinupristin/dalfopristin (0.5–4 µg/mL), rifampin (0.02–0.5 µg/mL), streptomycin (1–16 µg/mL), sulfamethoxazole (64–512 µg/mL), tetracycline (0.5–16 µg/mL), tiamulin (0.5–4 µg/mL), trimethoprim (2–32 µg/mL), and vancomycin (1–16 µg/mL). Briefly, approximately 5 × 10^5^ colony forming unit (cfu)/mL inoculums, prepared from overnight cultures, were inoculated on the minimum inhibitory concentration (MIC) panels and incubated at 35 °C for 20–24 h. *S. aureus* ATCC 25,923 was used as a reference strain. The MIC values were interpreted according to the Clinical and Laboratory Standards Institute [41] and the European Committee on Antimicrobial Susceptibility Testing [43] guidelines. 

### 3.3. Detection of Mutations 

The central loop of domain V of the 23S rRNA and the genes encoding ribosomal proteins L3 (*rplC*) and L4 (*rplD*) were amplified using primers, as described previously [17,32]. The nucleotide and amino acid sequences of *rplC*, *rplD*, and domain V of the 23S rRNA gene, for each of the isolates tested, were compared with those of the wild-type linezolid-susceptible *S. aureus* ATCC29213 strain (GenBank accession no. NZ_MOPB01000038.1). Analysis and comparison were performed using the basic local alignment search tool (BLAST) program (http://www.ncbi.nlm.nih.gov/BLAST) and ExPASY proteomics tools (http://www.expasy.ch/tools/#similarity).

### 3.4. Molecular Typing of LR S. aureus

The LR isolates were further characterized by multilocus sequence typing (MLST). Sequences of the PCR products were compared with sequences available on the MLST website for *S. aureus* [44]. *S. aureus* protein A (spa) typing was performed using the method described by Enright et al. [45], and the spa types were assigned using the Ridom Staph Type server (Ridom GmbH, Wurzburg, Germany) (www.spaserver.ridom.de). Additionally, the staphylococcal cassette chromosome mec (*SCCmec*) typing was carried out in all LR isolates that harbored the *mecA* gene using PCR [46]. The detection of genes encoding the virulence determinants such as Panton–Valentine leucocidin (PVL), leukotoxins (lukED), exfoliatins (*eta* and *etb*), toxic shock syndrome toxin 1 (*tsst1*), and staphylococcal enterotoxins (*sea*, *seb*, *sec*, *sed*, *see*, *seg*, *seh*, *sei*, *selj*, *sek*, *sell*, *sem*, *sen*, *seo*, *sep*, *seq*, and ser) was performed by PCR [47]. The isolates were also investigated for three genes (*scn*, *chp*, and *sak*) that represent components of the immune evasion cluster [48].

Pulsed-field gel electrophoresis (PFGE) analysis of *SmaI*-digested chromosomal DNA was performed to investigate clonality [49]. Briefly, chromosomal DNA sample plugs were digested with 50 U of *Sma*I (Takara Bio, Otsu, Japan) and separated by electrophoresis on 1.0% SeaKem Gold agarose (Lonza, Allendale, NJ, USA) in 0.5× Tris–borate–Ethylenediaminetetraacetic acid EDTA buffer at 14 °C for 20 h using a CHEF-Mapper (Bio-Rad, Hercules, CA, USA) with the following parameters: initial switch time, 5.3 s; final switch time, 34.9 s; angle, 120°; gradient, 6.0 V/cm; ramping factor, linear. Results were analyzed using Bionumerics software, version 4.0 (Applied Maths, Sint-Martens-Latem, Belgium), and relatedness was calculated using the unweighted pair-group method with arithmetic averages (UPGMA) algorithm, on the basis of the Dice similarity index.

## 4. Conclusions

The occurrence of linezolid resistance is still rare among *S. aureus* isolates from animal carcasses. Nevertheless, we detected the multi-resistance gene *cfr* and the novel phenicol exporter gene *fexA* among all the LR *S. aureus* isolated from pigs. Mutations in ribosomal proteins rplC and rplD were also detected in some of the strains. Resistant strains could be transmitted to humans through the food supply chain, subsequently limiting the treatment options for multidrug-resistant *S. aureus*. Therefore, frequent screening of pig carcasses, farmers, and slaughterhouse environments, as well as thorough cooking of pig meat, should be implemented to detect the emergence and persistence of toxigenic and LR *S. aureus* strains in order to prevent dissemination to humans.

## Figures and Tables

**Table 1:** Prevalence of linezolid resistance in *Staphylococcus aureus* isolated from food animal carcasses in South Korea from 2010 to 2017.

Year	% (No. of Linezolid-Resistant Isolates/No. of Isolates)			
	Cattle	Pig	Chicken	Total
2010	0 (0/39)	0 (0/70)	0 (0/81)	0 (0/190)
2011	0 (0/69)	0 (0/101)	0 (0/137)	0 (0/307)
2012	0 (0/76)	9.8 (12/122)	0 (0/201)	3 (12/399)
2013	0 (0/49)	1.7 (3/178)	0 (0/133)	0.8 (3/360)
2014	0 (0/62)	1.1 (2/182)	0 (0/168)	0.5 (2/412)
2015	0 (0/41)	2.5 (4/160)	0 (0/195)	1 (4/396)
2016	0 (0/29)	1.9 (3/158)	0 (0/77)	1.1 (3/264)
2017	0 (0/17)	0.9 (1/106)	0 (0/96)	0.5 (1/219)
Total	0 (0/382)	2.3 (25/1077)	0 (0/1088)	1.0 (25/2547)

**Table 2:** Characteristics of linezolid-resistant *S. aureus* isolated from pig carcasses.

Isolate	Year	Provinces	Farm ID	MIC (µg/mL)					Other Resistance Phenotype	Genetic Resistance Marker								MLST	*Spa Type*	*SCCmec* Type	Virulence Patterns	Pulso Type
				LNZ	CHL	CLI	TIA	SYN		*mecA*	*cfr*	*fexA*	*optrA*	*poxtA*	23S rRNA	*rplC*	*rplD*					
V02-12-023	2012	Gyeonggi	GG-1	8	>64	>4	>4	>4	ERY, GEN, KAN, PEN, TMP	-	+	+	-	-	WT	WT	WT	5	t002	-	*seg-sei-sem-sen-seo*	A
V02-12-027	2012	Chungnam	CN-1	8	>64	>4	>4	4	FOX, PEN, TET	+	+	+	-	-	WT	WT	WT	398	t034	V		ND
V04-12-005	2012	Chungnam	CN-2	16	>64	>4	>4	2	GEN, KAN, PEN, TET	-	+	+	-		WT	WT	WT	5	t002	-	*seg-sei-sem-sen-seo-lukED*	A
V08-12-002	2012	Gyeongbuk	GB-1	8	>64	>4	>4	>4	FOX, CIP, ERY, GEN, KAN, PEN, TET	+	+	+	-	-	WT	WT	WT	541	t034	V		ND
V13-12-013	2012	Gyeongbuk	GB-2	16	>64	>4	>4	4	GEN, KAN, PEN, TET	-	+	+	-	-	WT	WT	C353T	433	t318	-	*seg*	B
V14-12-001	2012	Chungnam	CN-3	8	>64	>4	>4	4	TET	-	+	+	-	-	WT	WT	C353T	433	t318	-	*seg*	B
V14-12-008	2012	Chungnam	CN-3	16	>64	>4	>4	4	FOX, ERY, PEN, TET	+	+	+	-	-	WT	WT	WT	541	t034	V		ND
V14-12-011	2012	Gyeonggi	GG-2	16	>64	>4	>4	2	FOX, ERY, PEN, TET	+	+	+	-	-	WT	WT	WT	541	t034	V		ND
V14-12-012	2012	Incheon	IC-1	8	>64	>4	>4	>4	FOX, ERY, PEN, TET	+	+	+	-	-	WT	WT	WT	541	t034	V		ND
V14-12-015	2012	Chungnam	CN-4	8	>64	>4	>4	>4	CIP, ERY, GEN, KAN, PEN, TET, TMP	-	+	+	-	-	WT	WT	WT	541	t034	-		ND
V14-12-016	2012	Chungnam	CN-5	16	>64	>4	>4	4	-	-	+	+	-	-	WT	WT	C353T	433	t318	-	*seg*	B
V14-12-017	2012	Gyeonggi	GG-3	16	>64	>4	>4	4	-	-	+	+	-	-	WT	WT	C353T	433	t318	-	*seg*	B
V04-13-019	2013	Chungbuk	CB-1	16	>64	>4	>4	4	PEN	-	+	+	-	-	WT	WT	WT	9	t337	-	*seg-sei-sem-sen-seo*	C
V04-13-032	2013	Chungnam	CN-6	16	>64	>4	>4	4	PEN	-	+	+	-	-	WT	WT	WT	9	t337	-	*seg-sei-sem-sen-seo*	C
V08-13-003	2013	Gyeongbuk	GB-3	8	>64	>4	>4	4	PEN	-	+	+	-	-	WT	WT	WT	5	t548	-	*seg-sei-sem-sen-seo-lukED*	A
V04-14-023	2014	Chungbuk	CB-2	8	>64	>4	>4	2	PEN	-	+	+	-	-	WT	G121A	WT	5	t002	-	*seg-sei-sem-sen-seo-lukED*	A-1
V14-14-006	2014	Chungnam	CN-7	8	>64	>4	>4	4	CIP, GEN, KAN, PEN	-	+	+	-	-	WT	WT	C353T	433	t318	-	*seg*	B
V02-15-007	2015	Gyeonggi	GG-4	8	>64	>4	>4	2	GEN, KAN, PEN	-	+	+	-	-	WT	WT	WT	2007	t8314	-	*seg-sei-sem-sen-seo*	D
V14-15-002	2015	Incheon	IC-2	8	>64	>4	>4	2	TET	-	+	+	-	-	WT	WT	C353T	433	t318	-	*seg*	B
V14-15-016	2015	Incheon	IC-3	8	>64	>4	>4	>4	FOX, ERY, PEN, TET	+	+	+	-	-	WT	WT	WT	541	t034	V		ND
V15-15-012	2015	Jeonnam	JN-1	8	>64	>4	>4	4	PEN	-	+	+	-	-	WT	WT	WT	9	t337	-	*seg-sei-sem-sen-seo*	C
V03-16-003	2016	Gangwon	GW-1	8	>64	>4	>4	4	GEN, KAN, PEN	*-*	*+*	+	-	-	WT	WT	WT	5	t002	-	*seg-sei-sem-sen-seo-lukED*	A
V06-16-007	2016	Jeonbuk	JB-1	8	>64	>4	>4	2	PEN, TET	*-*	*+*	+	-	-	WT	WT	WT	9	t899	-	*seg-sei-sem-sen-seo*	C-1
V14-16-004	2016	Gyeonggi	GG-5	8	>64	>4	>4	4	CIP, ERY, PEN, TET, TMP	*-*	*+*	+	-	-	WT	WT	WT	398	t1170	-		ND
V13-17-011	2017	Gyeongbuk	GB-4	8	64	>4	>4	4	-	-	+	+	-	-	WT	WT	C353T	433	t021	-	*seg*	B

Abbreviations: LNZ, linezolid; CHL, chloramphenicol; CLI, clindamycin; TIA, tiamulin; SYN, quinupristin/dalfopristin; FOX, cefoxitin; CIP, ciprofloxacin; ERY, erythromycin; GEN, gentamicin; KAN, kanamycin; PEN, penicillin; TET, tetracycline; TMP, trimethoprim; WT, wild type; MLST, multi-locus sequence type. SCCmec typing was performed for methicillin-resistant *Staphylococcus aureus* (MRSA) strains only.

## Supplementary Materials

The following are available online at https://www.mdpi.com/2079-6382/9/11/769/s1, Figure S1: *Sma I*-digested pulse-field gel electrophoresis patterns of linezolid-resistant *S. aureus* isolated from pig carcasses in Korea. Genomic DNA of ST398 and ST541 are not digested by *SmaI*, and hence pulsed-field gel electrophoresis (PFGE) patterns were not determined.
