# Supplementary figures and images for: Emergence of cfr-Mediated Linezolid Resistance in Staphylococcus aureus Isolated from Pig Carcasses
Source: Antibiotics (Basel). 2020 Nov 2;9(11):769. doi: 10.3390/antibiotics9110769 (PMC7692708; doi:10.3390/antibiotics9110769)

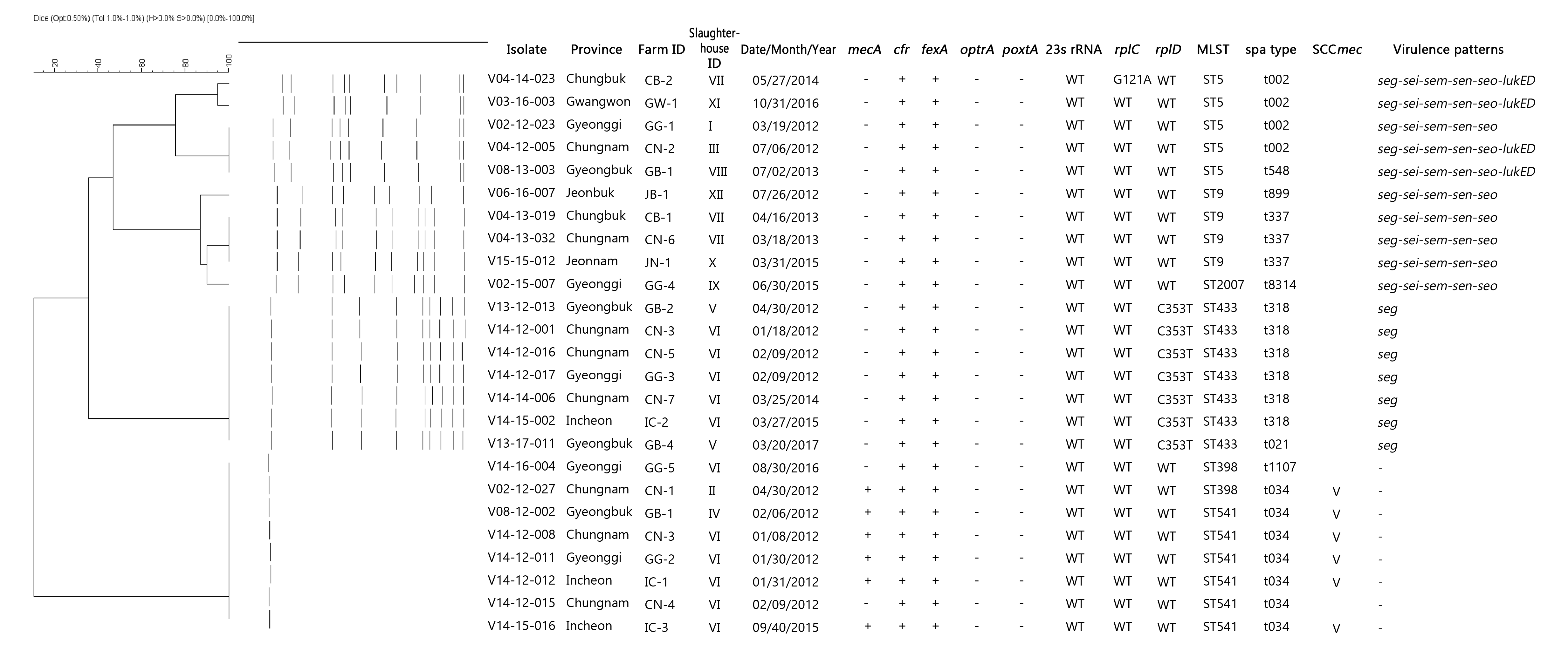

Supplement: Supplementary file 1 [file antibiotics-09-00769-s001.zip › antibiotics-972618-supplementary.TIF]
